# HIV-1 Infection Alters the Viral Composition of Plasma in Men Who Have Sex with Men

**DOI:** 10.1128/mSphere.00081-21

**Published:** 2021-05-05

**Authors:** Kai Liu, Yanpeng Li, Rong Xu, Yan Zhang, Chenli Zheng, Zhenzhou Wan, Hao Li, Zhengrong Yang, Xia Jin, Pei Hao, Jin Zhao, Chiyu Zhang

**Affiliations:** a Key Laboratory of Molecular Virology and Immunology, Institut Pasteur of Shanghai, Chinese Academy of Sciences, Shanghai, China; b University of Chinese Academy of Sciences, Shanghai, China; c Shanghai Clinical Research Center for Infectious Disease (HIV/AIDS), Shanghai Public Health Clinical Center, Fudan University, Shanghai, China; d Shenzhen Center for Disease Control and Prevention, Shenzhen, China; e Medical Laboratory of Taizhou Fourth People’s Hospital, Taizhou, China; Icahn School of Medicine at Mount Sinai

**Keywords:** MSM, plasma virome, HIV-1 infection, viral metagenome, pegivirus, anellovirus,

## Abstract

Altered gut virome and expanded abundance of certain viruses were found in HIV-1-infected individuals. It remains largely unknown how plasma virus composition changes during HIV-1 infection and antiretroviral therapy (ART). We performed viral metagenomic analysis on viral particles enriched from human plasma from 101 men who have sex with men (MSM) with or without HIV-1 infection and whether or not on ART and compared the differences in the plasma virome. An increased plasma viral abundance of main eukaryotic viruses was observed during HIV-1 infection in MSM, especially in AIDS patients (CD4^+^ T cell counts of <200). Anellovirus, pegivirus and hepatitis B virus (HBV) were the most abundant blood-borne viruses detected among MSM and HIV-1-infected individuals, and anellovirus and pegivirus were closely related to HIV-1 infection. High diversity of anelloviruses was found mostly in HIV-1-infected MSM, and their abundance was positively correlated with the HIV-1 viral load, but negatively correlated with both CD4^+^ T cell counts and CD4^+^/CD8^+^ ratio; in contrast, the abundance of pegivirus showed opposite correlations. ART usage could restore the plasma virome toward that of HIV-1-negative individuals. These data showed an expansion in abundance of certain viruses during HIV-1 infection, indicating the higher risk of shedding some blood-borne viruses in these individuals. These investigations indicate that both anellovirus and pegivirus may play certain roles in HIV disease progression.

**IMPORTANCE** Though an increasing number of studies have indicated the existence of an interaction between the virome and human health or disease, the specific role of these plasma viral components remains largely unsolved. We provide evidence here that an altered plasma virome profile is associated with different immune status of HIV-1 infection. Specific resident viruses, such as anellovirus and pegivirus, may directly or indirectly participate in the disease progression of HIV-1 infection. These results can help to determine their clinical relevance and design potential therapies.

## Abstract (precis)

Though an increasing number of studies have indicated the existence of an interaction between the virome and human health or disease, the specific role of these plasma viral components remains largely unsolved. We provide evidence here that an altered plasma virome profile is associated with different immune statuses of HIV-1 infection.

## INTRODUCTION

Viruses are the most abundant and variable components of the microbiome in human bodies (1, 2), and they could affect human health by direct interaction with host immunity or indirect interaction with other components of human microbiome. Previous studies showed that the human virome may change under different pathological conditions and immune status (3, 4). For example, altered viral composition of gut has been observed in cancer, diabetes, inflammatory bowel disease (IBD), and AIDS (5–8), and changes of a specific virome profile may be predictive of disease development (9).

An impaired immune system after HIV-1 infection can lead to substantial changes of the host residential microbial composition, including an increased diversity or abundance of gut viral and bacterial communities. For example, during HIV-1 infection, an expansion of adenoviruses in the gut has been demonstrated in association with a decrease in CD4^+^ T cell counts (10). Similarly, increased abundance of enteroviruses, including adenoviruses, was found to be associated with AIDS symptoms in simian immunodeficiency virus (SIV)-infected macaques (11). These data indicated a close relationship of particular viruses in immunodeficiency and AIDS-associated enteropathy. Though relatively less diverse than gut microbial communities, human plasma is major carrier of pathogenic viruses, as well as many commensal viruses (12). Previous studies found that anelloviruses, adenoviruses, human endogenous retrovirus (HERV), herpesviruses, papillomaviruses, polyomaviruses, and pegivirus could be present in the general population without specific disease status, indicating the complex sources and still undefined roles of most viruses in certain individuals (1). Early studies showed an altered plasma virome after HIV-1 infection, and an increased abundance of anellovirus and HERV was seen in AIDS patients (8, 13). Besides, specific plasma viral components were also associated with positive outcomes: for example, coinfection with pegivirus could inhibit HIV-1 replication and had a beneficial impact on patients’ survival (14, 15).

The population of men who have sex with men (MSM) has higher risk of infection by HIV-1 and other infectious viruses. The rate of new HIV-1 infection among this population has still been increasing in most countries over the past 20 years (16, 17). An estimated 1.7 million people were newly infected by HIV-1 in 2018, and 17% of them were MSM. Recent HIV-1 prevalence surveys in different MSM communities showed prevalence ranging from 4% to 15% (18). Using viral metagenomics, we characterized the plasma virome during HIV-1 infection of MSM, trying to determine a comprehensive profile of the circulating virome of these individuals, as well as how the plasma virome changes in response to HIV-1 infection, antiretroviral therapy (ART) usage, and immune status. This will help to understand the role of plasma virome in HIV-1 progression and identify potential viral biomarkers.

## RESULTS

### Participants.

A total of 101 MSM with or without HIV-1 infection and 20 HIV-1-negative non-MSM as a control were recruited. All individuals were between 23 and 42 years old. All MSM samples were divided into four groups: HIV-1-negative MSM (*n* = 22), HIV-1-infected ART-naive MSM with CD4 > 200 (*n* = 30) or CD4 < 200 (*n* = 20), and HIV-1-infected ART-treated MSM (*n* = 29). ART usage in the HIV-1-infected ART-treated MSM group lasted for at least 8 months (median = 38.7; interquartile range [IQR], 24.7 to 65.3), plasma HIV-1 viral loads were below the limit of detection (<20 copies/ml), and CD4^+^ T cell counts were above 200 cells/μl. HIV-1 viral load in the ART-naive groups ranged from 0.56 × 10^5^ to 11.35 × 10^5^ copies/ml and was negatively correlated with CD4^+^ T cell counts (Spearman’s *r* = −0.41, *P* = 0.0032) or CD4^+^/CD8^+^ ratio (Spearman’s *r* = −0.44, *P* = 0.0017) (Table 1; see Fig. S1A in the supplemental material).

**TABLE 1:** Cohort characteristics

Patient characteristic	Result for group^*a*^				
	Non-MSM	HIV-1^−^ MSM	HIV-1^+^ ART-naive MSM		ART-treated MSM (CD4 > 200)
			CD4 > 200	CD4 < 200	
No. of samples	20	22	30	20	29
Age (yr)	28 (24–35)	29.5 (25–38)	26 (23–30)	32 (27–36)	37 (30–42)
HIV-1 viral load (copies/ml)	−	<20^*b*^	1.03 ×10^5^ (0.56 × 10^5^–2.86 × 10^5^)	4.8 ×10^5^ (2.02 × 10^5^–11.35 ×10^5^)	<20^*b*^
T cell count (cells/μl)					
CD4^+^	−	−	325.5 (277–386)	84.5 (35.5–161)	527 (433–621)
CD8^+^	−	−	1,014 (681–1,272)	658 (595.5–829)	682 (521–935)
CD4^+^/CD8^+^ ratio	−	−	0.35 (0.28–0.46)	0.13 (0.06–0.20)	0.77 (0.59–1.04)
No. of mo on ART	−	−	−	−	38.7 (24.7–65.3)

a Data are shown as medians with interquartile ranges in parentheses. A minus sign (−) indicates not tested.

b The detection limit of the HIV-1 quantification kit used is 20 copies/ml.

10.1128/mSphere.00081-21.1 FIG S1 Correlation analysis of HIV-1 viral load with CD4^+^ T cell counts, CD8^+^ T cell counts, and CD4^+^/CD8^+^ ratio. (A) Correlation between peripheral circulating CD4^+^ T cell counts, CD8^+^ T cell counts, CD4^+^/CD8^+^ ratio, and HIV-1 viral load in HIV-1-infected ART-naive MSM. (B) Spearman's correlation between HIV-1 viral load and HIV-1 read count (normalized by RPM). Download FIG S1, TIF file, 0.5 MB. Copyright © 2021 Liu et al. 2021 Liu et al. https://creativecommons.org/licenses/by/4.0/ This content is distributed under the terms of the Creative Commons Attribution 4.0 International license.

### Viral composition in human plasma of MSM.

**FIG 1:**
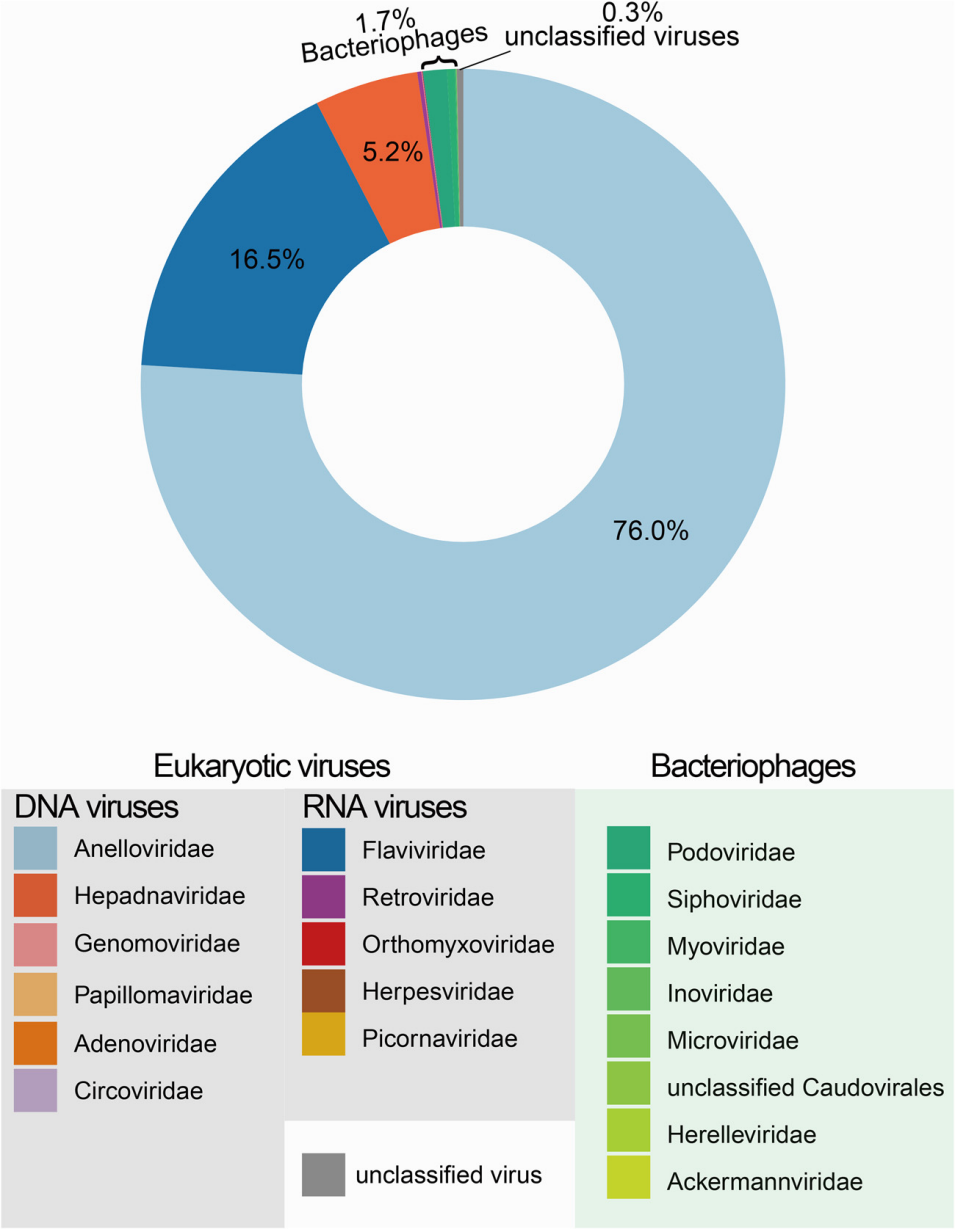
Viral metagenome composition from all individuals of this study. The donut chart shows the viral composition of the plasma virome from all individuals of this study. Eukaryotic viruses and bacteriophages are labeled in different colors.

All plasma samples were first processed through virus-like particle (VLP) enrichment and then subjected to the Illumina NovaSeq platform. On average, 67 million reads were obtained from each sample, and after low-quality and human genome reads were removed, 17 million reads from each sample were obtained for subsequent analyses. The majority of cleaned reads were unclassified (∼57%); viral reads accounted for ∼10% of cleaned reads (see Fig. S2A in the supplemental material). In general, the plasma virome of these participants were dominated by eukaryotic viruses (98.0% of total viral reads), followed by bacteriophages (1.7%) and unclassified viruses (0.3%) (see Table S1 in the supplemental material). Here, we focused on the main pathogenic and commensal human plasma viruses: in total, 11 eukaryotic viral families were detected, including DNA viruses *Anelloviridae*, *Hepadnaviridae*, *Genomoviridae*, *Papillomaviridae*, *Adenoviridae*, *Circoviridae*, and *Herpesviridae* and RNA viruses *Flaviviridae* (e.g., pegivirus and hepatitis C virus [HCV]), *Retroviridae*, *Orthomyxoviridae*, and *Picornaviridae* (Fig. 1; Table S1).

10.1128/mSphere.00081-21.2 FIG S2 Taxonomic composition of NGS results and correlation analysis of anellovirus and pegivirus with CD4^+^ T cell counts, CD8^+^ T cell counts, and CD4^+^/CD8^+^ ratio. (A) Taxonomic composition of different plasma samples as determined by NGS. Abundance is shown as reads per million (RPM); other terms represent unclassified eukaryotic reads or unknown reads. (B) Spearman’s correlation between anellovirus and CD8^+^ T cell counts, CD4^+^ T cell counts, and the CD4^+^/CD8^+^ ratio in ART-treated or ART-naive groups. (C) Spearman’s correlation between pegivirus and CD4^+^ T cell counts, CD8^+^ T cell counts, and CD4^+^/CD8^+^ ratio in ART-treated or ART-naive groups. Download FIG S2, TIF file, 1.4 MB. Copyright © 2021 Liu et al. 2021 Liu et al. https://creativecommons.org/licenses/by/4.0/ This content is distributed under the terms of the Creative Commons Attribution 4.0 International license.

10.1128/mSphere.00081-21.3 TABLE S1 All the main viral families detected and number of sequencing reads. Download Table S1, DOCX file, 0.01 MB. Copyright © 2021 Liu et al. 2021 Liu et al. https://creativecommons.org/licenses/by/4.0/ This content is distributed under the terms of the Creative Commons Attribution 4.0 International license.

**FIG 2:**
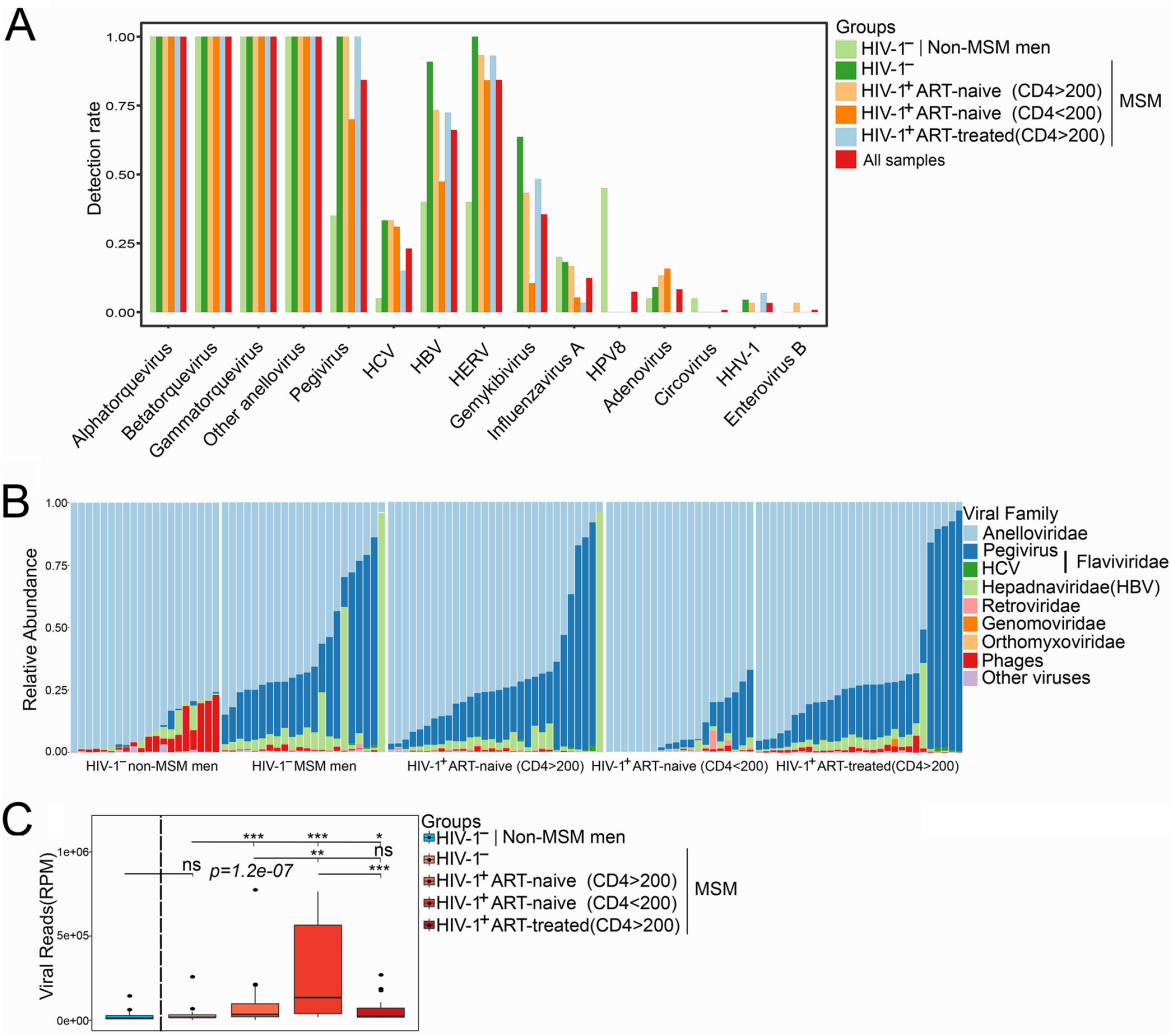
Distribution of the main plasma viruses in different groups. (A) Positive rate for main viruses from all individuals. (B) Family-level taxonomic composition of the plasma virome; the relative abundance of the top 6 virus families is shown in the bar plot. (C) Comparison of viral abundance between different groups, shown as reads per million (RPM).

HIV-1 reads were identified in 65 out of 79 HIV-1-positive samples and positively correlated with HIV-1 viral load (Spearman’s *r* = 0.55, *P* = 5.7e−05) (Fig. S1B), reflecting consistent results from both next-generation sequencing (NGS) and quantitative reverse-transcription PCR (RT-qPCR) for HIV-1 detection. Besides HIV-1, the most frequently detected viruses from these individuals included anellovirus, pegivirus, hepatitis B virus (HBV), HCV, and HERV, whose detection rates were 100% (121/121), 84.3% (102/121), 43.8% (53/121), 23.1% (28/121), and 84.3% (102/121), respectively. Other viruses included papillomavirus (9/121 [7.4%]), influenza virus (15/121 [12.4%]), alphaherpesvirus (human herpesvirus 1 [HHV-1]) (4/121 [3.3%]), adenovirus (10/121 [8.3%]), and gemykibivirus (43/121 [35.5%]). Circovirus or enterovirus B was only detected in a single individual (Fig. 2A; see Table S2 in the supplemental material).

10.1128/mSphere.00081-21.4 TABLE S2 Detection rate of plasma viruses in different groups. Download Table S2, DOCX file, 0.01 MB. Copyright © 2021 Liu et al. 2021 Liu et al. https://creativecommons.org/licenses/by/4.0/ This content is distributed under the terms of the Creative Commons Attribution 4.0 International license.

### HIV-1 infection reshaped the plasma virome of MSM.

Compared to non-MSM individuals, MSM did not show obvious changes in overall plasma viral reads; however, HIV-1 infection significantly increased the abundance of plasma viral reads. AIDS patients (CD4 < 200) had the highest abundance of viral reads (excluding HIV-1 reads). Even though the viral abundance of individuals receiving ART (CD4 > 200) was higher than that of HIV-1-negative MSM, ART successfully controlled the shedding of different viruses into plasma (Fig. 2C).

When specific viral components were examined, HIV-1 infection led to a decrease in phage abundance and increase in eukaryotic virus abundance in MSM (especially pegivirus), compared to non-MSM (Fig. 2B). MSM and HIV-1-infected individuals displayed similar plasma viral profiles, with anellovirus being dominant in almost all individuals, followed by pegivirus and HBV. ART usage could restore the virome profile toward that of HIV-1-negative individuals. There was no difference in detection rate of anellovirus among different groups, while HIV-1-infected MSM with CD4 < 200 had relatively lower detection rate for several viruses, including pegivirus, HBV, HCV, and HERV compared to other MSM (Fig. 2A).

### Association of anellovirus with the progress of HIV-1 infection.

**FIG 3:**
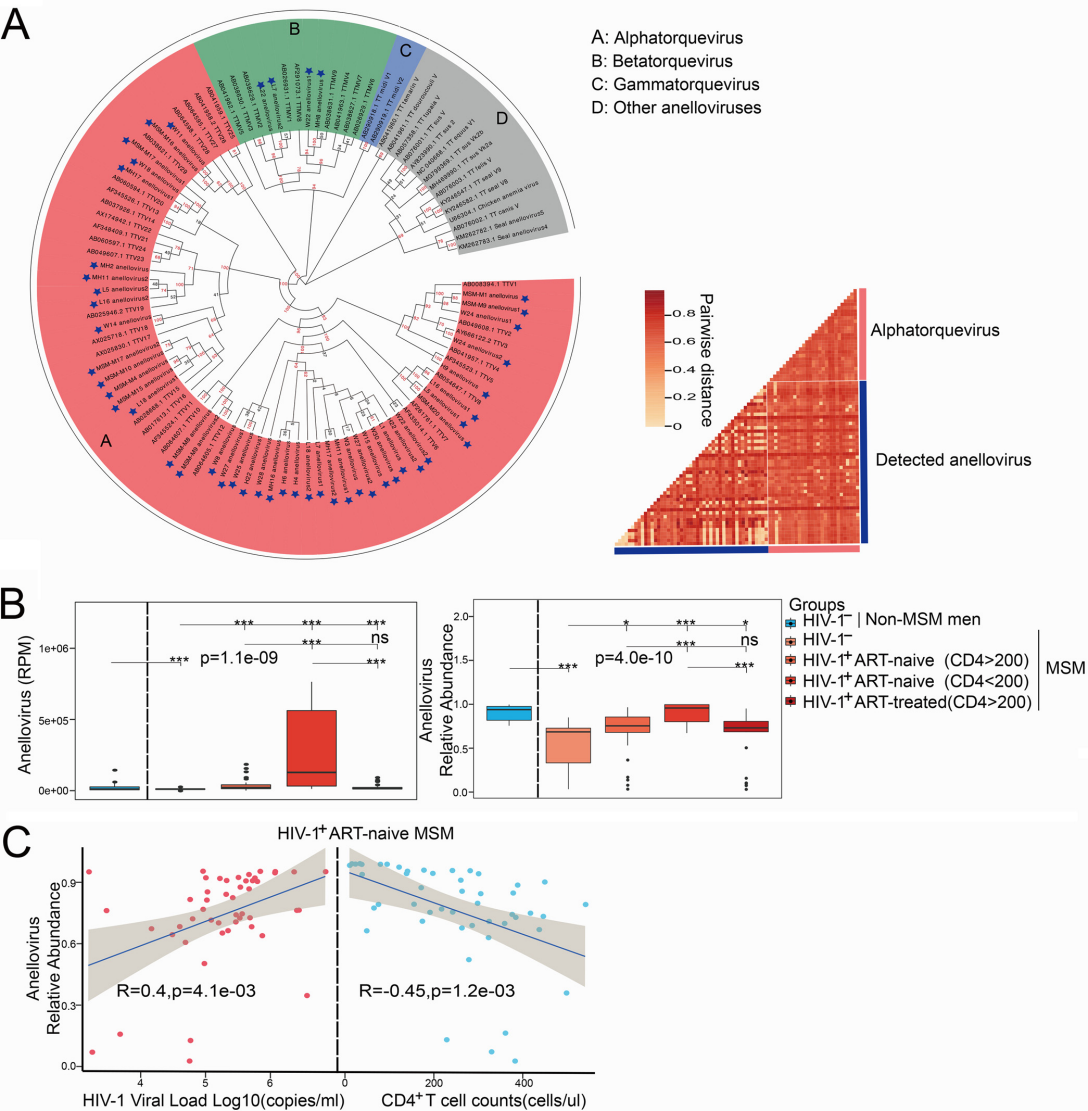
Associations of anellovirus with HIV-1 infection and CD4^+^ T cell counts. (A, left) Phylogenetic tree of anellovirus; newly discovered anelloviruses are labeled with blue stars. The sample source of each anellovirus is indicated as follows: MSM-M, HIV-1-negative non-MSM; MH, HIV-1-negative MSM; W, HIV-1-infected ART-naive MSM (CD4 > 200); L, AIDS patients (CD4 < 200); H, HIV-1-infected ART-treated MSM (CD4 > 200). The number at the end of the name represents the number of full-length ORF1 genes obtained. (Right) The heat map represents the pairwise distance of anelloviruses. (B, left) Read count (normalized by RPM) of pegivirus in samples from different groups. (Right) Relative abundance of pegivirus in samples from different groups. (C) Spearman's correlation between anellovirus and HIV-1 viral load (left) and CD4^+^ T cell counts (right) in HIV-1-infected ART-naive MSM.

As anellovirus dominated the plasma virome, we further analyzed its changes and diversity in different groups. Compared to non-MSM, MSM had a lower abundance of anellovirus. HIV-1 infection increased the abundance of anellovirus, with AIDS patients (CD4 < 200) showing the highest abundance. In addition, ART could decrease the abundance of anellovirus (Fig. 3B). Importantly, anellovirus had a significantly negative correlation with CD4^+^ T cell counts (Spearman’s *r *= 0.4, *P* = 0.0041) and CD4^+^/CD8^+^ ratio (Spearman’s *r* = −0.44, *P* = 1.4e−03), and a positive correlation was observed between anellovirus and HIV-1 viral load. After ART, those correlations disappeared (Fig. 3C; and Fig. S2B in the supplemental material).

All three main genera (alpha, beta, and gamma) of anelloviruses could be annotated using reads and partial contigs. In total, we obtained 44 alphatorqueviruses and 4 betatorqueviruses with full-length open reading frame 1 (ORF1) sequences. Phylogenetic analysis based on these 48 new anelloviruses and other references showed that these anelloviruses were highly divergent from each other, with a mean pairwise distance of about 0.69 (Fig. 3A).

### Association of pegivirus with the progress of HIV-1 infection.

**FIG 4:**
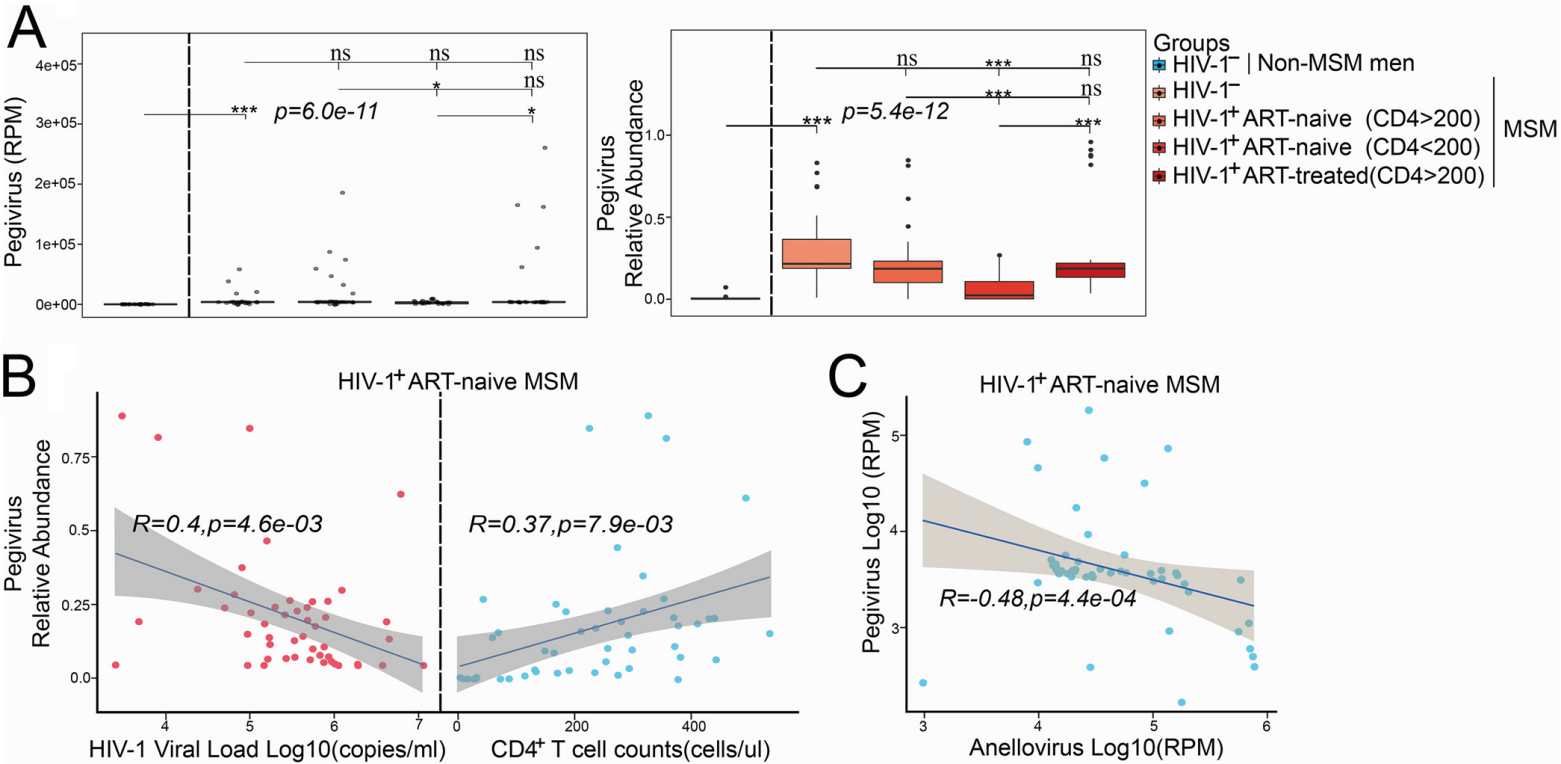
Associations of pegivirus with HIV-1 infection and CD4^+^ T cell counts. (A, left) Read count (normalized by RPM) of pegivirus in samples from different groups. (Right) Relative abundance of pegivirus in samples from different groups. (B) Spearman's correlation between pegivirus and HIV-1 viral load (left) and CD4^+^ T cell counts (right) in HIV-1-infected ART-naive MSM. (C) Spearman's correlation between anelloviridae and pegivirus read counts in HIV-1-infected ART-naive MSM.

*Pegivirus C* was the only species from *Pegivirus* genus identified from these participants. MSM had a higher abundance of pegivirus than non-MSM, and HIV-1 infection slightly decreased the abundance of pegivirus. The lowest abundance of pegivirus was observed in AIDS patients (CD4 < 200), even though they had the highest overall plasma viral abundance (Fig. 4A). To explore whether pegivirus changes along the HIV-1 disease progression, we compared the relative abundances of pegivirus among HIV-1-infected MSM with different clinical indexes. In HIV-1-infected ART-naive MSM, pegivirus abundance showed a positive correlation with CD4^+^ T cell counts (Spearman’s *r *= 0.37, *P* = 0.0079) and CD4^+^/CD8^+^ ratio (Spearman’s *r *= 0.41, *P* = 0.038) and a negative correlation with HIV-1 viral load (Spearman’s *r *= 0.4, *P* = 0.0046) (Fig. 4B; Fig. S2C). No significant correlation between pegivirus and any clinical indexes (CD4^+^ T cell counts, CD8^+^ T cell counts, and CD4^+^/CD8^+^ ratio) was found in MSM receiving ART (Fig. S2C). A negative correlation was observed between the abundances of pegivirus and anellovirus (Fig. 4C).

## DISCUSSION

In this study, we reported the total plasma virome in MSM and its change during HIV-1 infection. Overall, same as in previous reports, the most frequently found viruses in these populations are blood-borne viruses, such as anellovirus, pegivirus, HBV, HCV, and HERV. Except for anellovirus, HIV-1 infection in MSM led to higher positive rate for pegivirus, HBV, HCV, as well as HERV, indicating a higher risk of infection by other viruses in this Chinese population, due possibly to unsafe sex or impaired immune status. An increased plasma viral load was observed after HIV-1 infection, which was associated with lower CD4^+^ T cell counts. Besides HIV-1 infection, ART could also reduce the shedding of several other viruses into the plasma.

Anelloviruses constitute a diverse group of species and are highly prevalent in the healthy population. Increased anellovirus abundance was observed after immunosuppressive therapy and organ transplantation (9, 19), and it is considered a potential indicator for immune status or disease progression (20). As the most abundant viruses in the blood, a recent study showed anellovirus was almost ubiquitous in the general population (21). Even though the prevalence of anellovirus was also the same here in MSM and after HIV-1 infection, its abundance greatly increased after HIV-1 infection and was negatively correlated with CD4^+^ T cell counts, which could be restored by ART. Our data again highlighted the potential use of anellovirus as a predictor of disease pathophysiology or immune status. However, decreased anellovirus abundance was observed in MSM compared to the non-MSM group, probably due to a bloom in pegivirus. Pegivirus is an RNA virus that belongs to the *Flaviviridae* family and commonly causes persistent infection (22, 23). The global prevalence of pegivirus in healthy blood donors ranges from 1% to 9% among different regions (24), but in HIV-1-infected populations, the prevalence is higher—up to more than 80% (25, 26). Recent studies in China showed that the prevalences of pegivirus in intravenous drug users (IDUs) and MSM were ∼30% and 18.3%, respectively (25, 27), indicating a higher risk of transmission through blood. The increased abundance of plasma pegivirus in MSM here simply reflected the higher infection risk for them and raised concerns that unsafe blood sharing or transfusion may further increase the transmission of pegivirus among the MSM population. Unlike anellovirus, AIDS patients had lowest abundance of pegivirus, and the shedding of pegivirus in AIDS patients was inversely associated with the host’s immune status. Previous studies reported that pegivirus inhibited HIV-1 replication, and coinfection with pegivirus in HIV-1-infected individuals could promote survival (14, 15, 28). Anellovirus and pegivirus are the main components of the plasma virome with the highest abundance: their inverse correlation indicates that both viruses’ persistent infections may have different modulation effects on the immune system, and they could be potential indicators of the disease progression of HIV-1 infection. However, the causal relationship of both viruses and disease pathophysiology or immune status remains to be elucidated.

Other viruses were also detected in the plasma, reflecting their complex sources. For example, all participants were sampled without clear signs of respiratory symptoms: the detection of influenza virus A in 15 individuals could be the results of asymptomatic infections of these people, since influenza virus infections with no clear symptoms are quite common (29). Gemykibivirus belongs to a divergent group of single-stranded DNA viruses, and it was detected in some MSM with or without HIV-1 infection. Several studies showed its presence/enrichment in HIV-1-infected people and also patients with other diseases (30–33), which suggested this virus could be associated with human disease (34). Even though more evidence is still needed, the detection of gemykibivirus here raises question about its pathogenic role in human. That 9 of 10 adenovirus cases and all human herpesvirus 1 (HHV-1 [4/4]) and human enterovirus B (HEV-B [1/1]) cases were detected in MSM and HIV-1-infected individuals indicates the increased risk of infections of different transmissible viruses in these populations. Papillomaviruses can persist in peripheral blood mononuclear cells (PBMCs), and healthy blood donors from a previous study showed 8 to 15% positive rate for the HPV genome (35), so the positivity for HPV in the healthy group could represent a general distribution of this virus in these investigated individuals.

Even though this study compared the plasma virome changes in different groups, this is a cross-sectional study, and further research on dynamics of the virome, especially the abundance changes of anellovirus and pegivirus along with disease progression, is needed to validate the clinical implications of them. Another limitation is that we used random genome amplification to enrich virus genomes, which may magnify the real abundance of some viruses such as anellovirus.

In summary, we systematically compared plasma virome in MSM with or without HIV-1 infection and compared the virome changes at different CD4^+^ T cell counts. Altered plasma viral composition was observed in MSM and those with HIV-1 infection. Overall, anellovirus shedding was proportional to CD4^+^ T cell counts and positively correlated with HIV-1 viral load; opposite correlations were observed between the clinical parameters of HIV infection and pegivirus abundance, suggesting these two types of viruses may have different roles in the regulation of immune status. Further functional studies are needed to investigate the specific functions of both viruses in MSM and patients with HIV-1 infection.

## MATERIALS AND METHODS

### Ethics statement.

This study was approved by the Ethics Committees of Shenzhen Center for Disease Control and Prevention (Shenzhen CDC) and Institut Pasteur of Shanghai and complied with all relevant ethical regulations. Oral or written informed consent was obtained from volunteers before sample collection.

### Participants, sampling and viral metagenomics processing.

All participants were recruited from Shenzhen CDC, China (Table 1). At least 2 ml blood was collected from each participant, and plasma was separated within 24 h at Shenzhen CDC, China. All plasma samples were stored at –80°C until use. Quantification of HIV-1 viral load was done by quantitative reverse transcription-PCR (RT-qPCR; diagnostic kit from Da An Gene Co., Ltd., of Sun Yat-Sen University). Virus-like particle (VLP) enrichment and nucleic acid preamplification were performed as previously described (36). Plasma samples were centrifuged at 12,000 rpm at 4°C for 10 min, and filtrates were passed through sterile 0.45-μm-pore filters (Corning, USA) to reduce the background of human and bacterial cells. Filtered samples were incubated with 20 U Benzonase (Merck, Germany), 15 U Turbo DNase (Life Technologies, USA), and 20 U RNase I (Promega, USA) to digest free nucleic acids (NAs) at 37°C for 1.5 h, 20 mM EDTA (Sigma-Aldrich, Germany) was added, and the mixture was incubated at 65°C for 10 min to terminate the digestion. Viral nucleic acids were extracted with the QIAamp miniElute virus kit (Qiagen, Germany). Total RNA extracts were reverse transcribed using the SuperScript III kit with a mixture of random hexamer primers and oligo(dT) primers. The cDNA was treated with RNase H (New England BioLabs, Inc., USA) prior to whole-genome amplification using a multiple annealing and looping-based amplification cycle (MALBAC) single-cell DNA Quick-Amp kit (37) (Yikon Genomics, China). Amplified DNA was sheared to an average fragment length of 250 bp using Covaris E210 (Covaris, USA). Sheared DNA was purified and used for Illumina library construction using NEBNext Ultra II DNA (New England BioLabs, Inc., USA). The sequencing libraries were quantified by Qubit3.0 (Invitrogen, USA). Sequencing was done on the Illumina Nova-seq platform (Illumina, USA) with 2 × 150-bp paired reads, yielding an average of 67 million reads per library.

### Bioinformatic analysis.

Sequencing data were demultiplexed using Illumina software to generate FastQ files for each sample. The raw data of NGS were first cleaned by Cutadapt v.1.18 and Trimmomatic v.0.38 (38) by removing Illumina sequencing adaptor and low-quality sequences (SLIDINGWINDOW:4:20 MINLEN:50). Human- and bacterium-derived sequences were filtered out by Bowtie2 v.2.3.4.3 (39). The remaining high-quality reads were *de novo* assembled by Megahit v.1.1.3 (40). Assembled contigs were dereplicated and clustered at 95% identity using Vsearch v.2.10.4 (41). After low-complexity contigs were removed, high-quality contigs with a length of more than 500 nucleotides (nt) were further filtered for human and bacterial sequences. BLASTX from DIAMOND v.0.9.24 (42) was used to search against the NCBI nonredundant protein sequence (nr) database to annotate viral contigs; the remaining contigs were mapped to the NCBI nucleotide sequence (nt) database by BLASTN (43, 44). Furthermore, cleaned reads were mapped to viral contigs by BLASTN. Viral reads were first normalized by reads per million (RPM), and then abundance was calculated.

### Phylogenetic tree construction.

The amino acid sequences of the anellovirus ORF1 gene were extracted from all anellovirus contigs using NCBI’s orfFinder tool. Reference ORF1 sequences of anelloviruses were downloaded from the NCBI’s GenBank database. All ORF1 sequences were aligned using MUSCLE (45), and the resulting alignment was used to construct a neighbor-joining phylogenetic tree with a *p*-distance model and 1,000 bootstraps by MEGA X (46). The tree was visualized and modified with evolview (47).

### Statistics.

Comparative statistical analyses between two groups were performed using the Mann-Whitney-Wilcoxon test. Comparative statistical analyses of three or more groups were performed using the Kruskal-Wallis *H* test. As shown on the figures, differences were considered significant at *P* < 0.001 (***), *P* < 0.01 (**), or *P* < 0.05 (*), and “ns” indicates no statistically significant differences (*P* > 0.05). For all studies, data are presented as representative of one independent experiment. All statistical analyses were performed by RStudio v.3.8.

### Data availability.

Sequencing data have been uploaded to the Sequence Read Archive (SRA) database under accession no. PRJNA634526.
