# Supplementary figures and images for: HIV-1 Infection Alters the Viral Composition of Plasma in Men Who Have Sex with Men
Source: mSphere. 2021 May 5;6(3):e00081-21. doi: 10.1128/mSphere.00081-21 (PMC8103983; doi:10.1128/mSphere.00081-21)

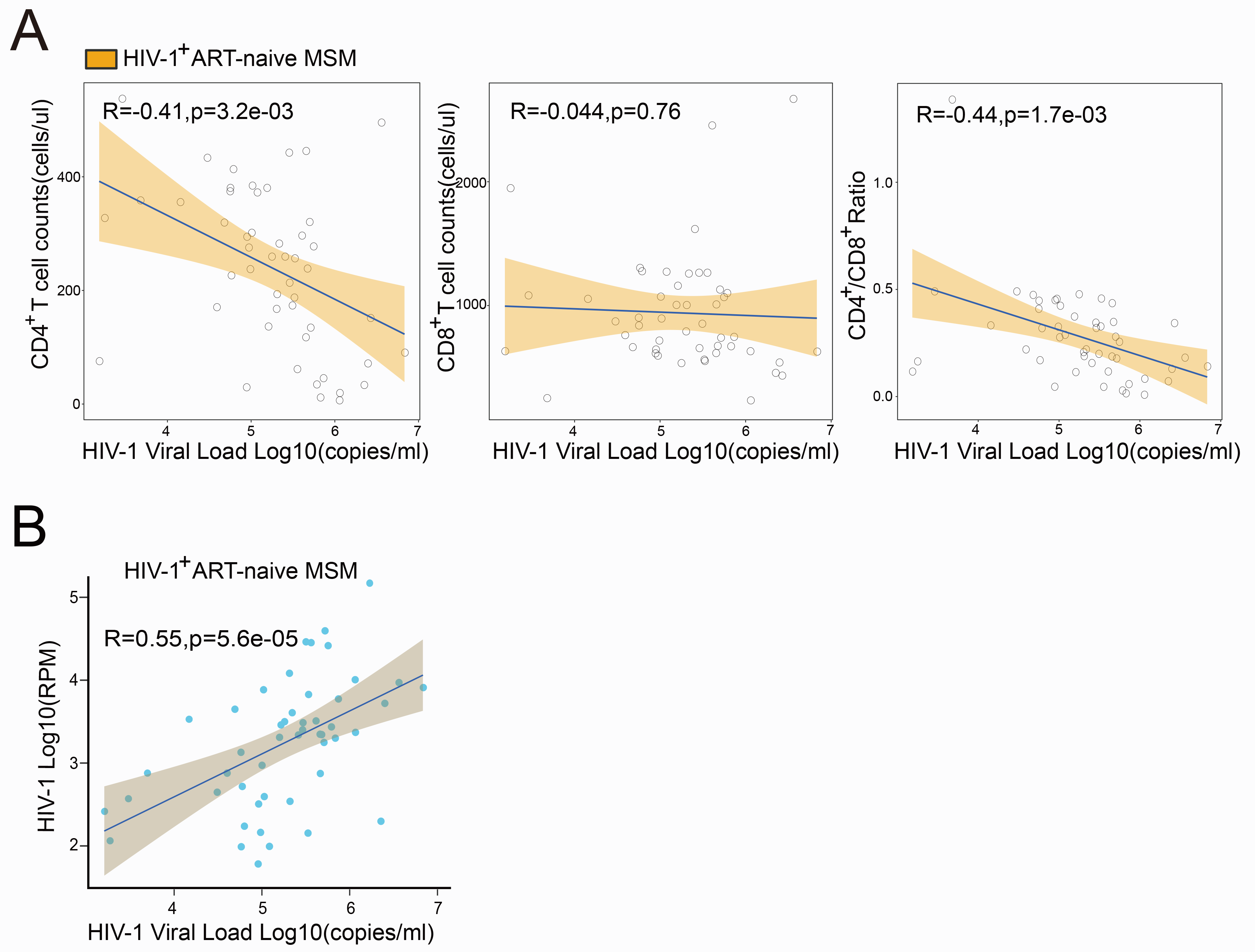

Supplement: FIG S1 [file mSphere.00081-21-sf001.tif]

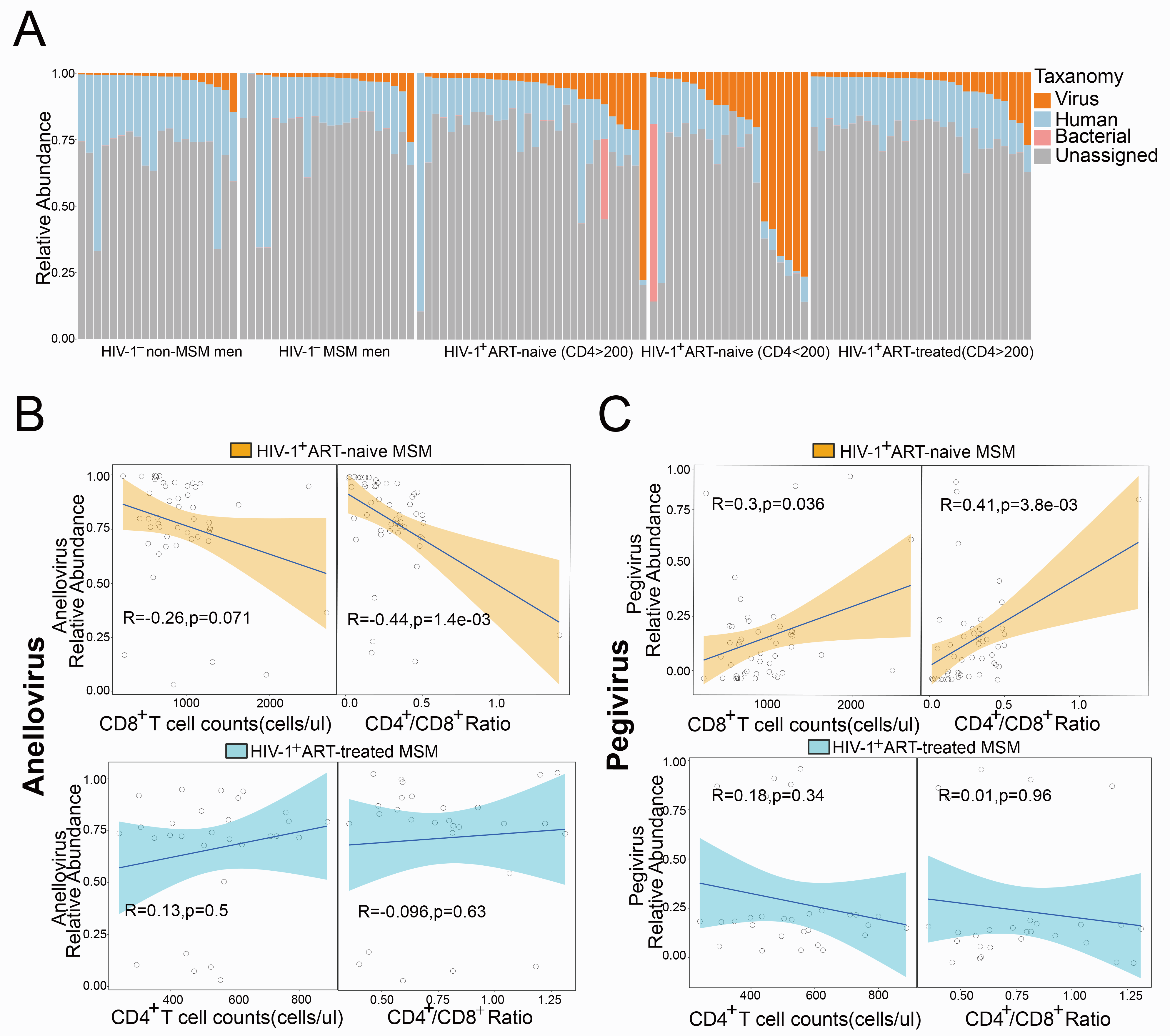

Supplement: FIG S2 [file mSphere.00081-21-sf002.tif]
