# Supplementary material for: HIV-1 Infection Alters the Viral Composition of Plasma in Men Who Have Sex with Men
Source: mSphere. 2021 May 5;6(3):e00081-21. doi: 10.1128/mSphere.00081-21 (PMC8103983; doi:10.1128/mSphere.00081-21)
Supplement: TABLE S1 [file mSphere.00081-21-st001.docx]

| Viral types | Family level | Reads number | Percent (%) |
| --- | --- | --- | --- |
| Eukaryotic viruses | Anelloviridae | 88238106 | 75.97298021 |
|  | Flaviviridae | 19201158 | 16.53219072 |
|  | Hepadnaviridae | 6062102 | 5.219467826 |
|  | Retroviridae | 247901 | 0.213442679 |
|  | Genomoviridae | 39583 | 0.03408095 |
|  | Orthomyxoviridae | 22148 | 0.019069421 |
|  | Papillomaviridae | 8417 | 0.007247034 |
|  | Adenoviridae | 4057 | 0.003493076 |
|  | Circoviridae | 3587 | 0.003088406 |
|  | Herpesviridae | 647 | 0.000557067 |
|  | Picornaviridae | 70 | 6.027E-05 |
| Phages | Podoviridae | 1374472 | 1.183419939 |
|  | Siphoviridae | 458410 | 0.394690859 |
|  | Myoviridae | 64552 | 0.055579251 |
|  | Inoviridae | 26762 | 0.023042073 |
|  | Microviridae | 6828 | 0.005878906 |
|  | unclassified Caudovirales | 3074 | 0.002646713 |
|  | Herelleviridae | 2193 | 0.001888172 |
|  | Ackermannviridae | 447 | 0.000384867 |
| Unclassified | Unclassified virus | 379549 | 0.326791564 |
