# Supplementary material for: HIV-1 Infection Alters the Viral Composition of Plasma in Men Who Have Sex with Men
Source: mSphere. 2021 May 5;6(3):e00081-21. doi: 10.1128/mSphere.00081-21 (PMC8103983; doi:10.1128/mSphere.00081-21)
Supplement: TABLE S2 [file mSphere.00081-21-st002.docx]

| Eukaryotic viruses, detection rate^a^ | Non-MSM men | | HIV-1(-) MSM | | HIV-1(+) MSM ART-naïve (CD4>200) | HIV-1(+) MSM ART-naïve (CD4<200) | MSM with ART-treated (CD4>200) | Total |
| --- | --- | --- | --- | --- | --- | --- | --- | --- |
| Alphatorquevirus | 20/20 | 22/22 | | 30/30 | | 20/20 | 29/29 | 121/121(100%) |
| Betatorquevirus | 20/20 | 22/22 | | 30/30 | | 20/20 | 29/29 | 121/121(100%) |
| Gammatorquevirus | 20/20 | 22/22 | | 30/30 | | 20/20 | 29/29 | 121/121(100%) |
| Other and unclassified anellovirus | 20/20 | 22/22 | | 30/30 | | 20/20 | 29/29 | 121/121(100%) |
| pegivirus | 7/20 | 22/22 | | 30/30 | | 14/20 | 29/29 | 102/121(84.3%) |
| HCV | 1/20 | 7/22 | | 10/30 | | 6/20 | 4/29 | 28/121(23.1%) |
| HBV | 8/20 | 21/22 | | 22/30 | | 9/20 | 21/29 | 81/121(67.9%) |
| HERV | 8/20 | 22/22 | | 28/30 | | 17/20 | 27/29 | 102/121(84.3%) |
| Gemykibivirus | 0/20 | 14/22 | | 13/30 | | 2/20 | 14/29 | 43/121(35.5%) |
| Influenzavirus A | 4/20 | 4/22 | | 5/30 | | 1/20 | 1/29 | 15/121(12.4%) |
| HPV8 | 9/20 | 0/22 | | 0/30 | | 0/20 | 0/29 | 9/121(7.4%) |
| Adenovirus | 1/20 | 2/22 | | 4/30 | | 3/20 | 0/29 | 10/121(8.3%) |
| Circovirus | 1/20 | 0/22 | | 0/30 | | 0/20 | 0/29 | 1/121(0.8%) |
| HHV-1 | 0/20 | 1/22 | | 1/30 | | 0/20 | 2/29 | 4/121(3.3%) |
| Enterovirus B | 0/20 | 0/22 | | 1/30 | | 0/20 | 0/29 | 1/121(0.8%) |

^a^Abbreviations in the table: HCV, hepatitis C virus; HBV, hepatitis B virus; HERV, human endogenous retroviruses; HPV8, human papillomavirus type 8; HHV-1, human herpesvirus 1.
